# Building Complex Autologous Breast Reconstruction Program: A Preliminary Experience

**DOI:** 10.3390/jcm12216810

**Published:** 2023-10-27

**Authors:** Min-Jeong Cho, Christopher A. Slater, Roman J. Skoracki, Albert H. Chao

**Affiliations:** Department of Plastic and Reconstructive Surgery, The Ohio State University Wexner Medical Center, Columbus, OH 43210, USA; christopher.slater@osumc.edu (C.A.S.); roman.skoracki@osumc.edu (R.J.S.); albert.chao@osumc.edu (A.H.C.)

**Keywords:** breast reconstruction, free flap, microsurgery, deep inferior epigastric perforator flap, profunda artery perforator flap, lumbar artery perforator flap, four-flap, complex autologous breast reconstruction, co-surgeon

## Abstract

Autologous breast reconstruction is an increasingly popular method of reconstruction for breast cancer survivors. While deep inferior epigastric perforator (DIEP) flaps are the gold standard, not all patients are ideal candidates for DIEP flaps due to low BMI, body habitus, or previous abdominal surgery. In these patients, complex autologous breast reconstruction can be performed, but there is a limited number of programs around the world due to high technical demand. Given the increased demand and need for complex autologous flaps, it is critical to build programs to increase patient access and teach future microsurgeons. In this paper, we discuss the steps, pearls, and preliminary experience of building a complex autologous breast reconstruction program in a tertiary academic center. We performed a retrospective chart review of patients who underwent starting the year prior to the creation of our program. Since the start of our program, a total of 74 breast mounds have been reconstructed in 46 patients using 87 flaps. Over 23 months, there was a decrease in median surgical time for bilateral reconstruction by 124 min (*p* = 0.03), an increase in the number of co-surgeon cases by 66% (*p* < 0.01), and an increase in the number of complex autologous breast reconstruction by 42% (*p* < 0.01). Our study shows that a complex autologous breast reconstruction program can be successfully established using a multi-phase approach, including the development of a robust co-surgeon model. In addition, we found that a dedicated program leads to increased patient access, decreased operative time, and enhancement of trainee education.

## 1. Introduction

Breast cancer is the most common cancer diagnosis in the United States, affecting one in eight women [1]. About 36% of breast cancer patients undergo mastectomies as a treatment, and 25% of these patients elect to have autologous-based reconstruction [2,3]. Autologous-based breast reconstruction uses a patient’s own tissue, typically from the patient’s abdomen, to restore the patient’s whole breast after mastectomy. It offers several advantages over an implant-based reconstruction, including aesthetically pleasing, natural feeling, and long-lasting breasts with higher patient satisfaction rates [4]. Living tissue transfer using deep inferior epigastric perforator (DIEP) flaps from the abdomen is the gold standard of abdominally based autologous breast reconstruction [5]. In this procedure, the soft tissue and fat from the abdomen are transferred to the patient’s chest, and microanastomosis is performed between the chest recipient vessels and the donor’s vessels. Recently, there has been a significant increase in the rate of autologous breast reconstruction due to the concerns of breast implant-associated anaplastic large cell lymphoma (BIA-ALCL) in textured implants and breast implant illnesses [6,7,8]. These concerns led to a 112% increase in the number of autologous-based reconstructions from 2009 to 2016 [9,10].

With the increased popularity and interest in autologous-based breast reconstruction, microsurgeons developed different reconstructive techniques for patients who are not candidates for abdominally based autologous reconstruction due to previous abdominoplasty, paucity of tissue, and patient preference [11,12]. Techniques such as stacked flaps, thigh-based flaps, and trunk-based flaps have been described to expand options for autologous breast reconstruction [13]. However, these options are technically demanding procedures that require technical expertise, longer operative time, and a team of microsurgeons [14]. Despite its disadvantages, complex autologous flaps offer unique autologous options for patients who were previously denied this reconstructive option due to tissue deficiencies and underwent multiple revisionary procedures due to a lack of available microsurgeons who can perform these procedures. 

Given the increased demand for autologous breast reconstruction and the growing need for complex autologous flaps, it is critical to build programs that will offer this unique option for breast cancer patients and teach future microsurgeons to ultimately increase access for patients. In this paper, we discuss the steps, pearls, and pitfalls of building a program that offers complex autologous breast reconstruction in a tertiary academic center. We will review various options for complex autologous reconstruction and phases of building the program and present preliminary data to show the successes and challenges we have faced in building a complex autologous program for breast reconstruction.

### 1.1. Types of Complex Autologous Breast Reconstruction

#### 1.1.1. Surgical Techniques: DIEP Flaps

In autologous breast reconstruction, the gold standard is the deep inferior epigastric perforator (DIEP) flap, accounting for nearly 70% of autologous reconstructions [3]. This flap was first described in 1992 by Allen and Treece. In this flap, the hemiabdomen is harvested, and the deep inferior epigastric vessels, which normally supply the inferior portion of the rectus abdominus muscle, serve as its pedicle. In contrast to the transverse rectus abdominus myocutaneous (TRAM) flap, DIEP flaps spare the majority of the rectus abdominus muscle, decreasing complications at the donor site [5,15]. A reported average DIEP weight is 681 g with a range of 284 g–1504 g [16]. The average deep inferior epigastric artery is from 2 to 3 mm in diameter, with veins between 2 and 3.5 mm in diameter [5]. DIEP perforator flaps are the preferred flaps due to their low donor-site morbidity, robust vascularity, and ample volume. Yet, there are limitations to this technique, including past abdominal surgeries, inadequate abdominal fat, and poor DIEP perforators [17].

#### 1.1.2. Surgical Techniques: APEX Flaps

The APEX (abdominal perforator exchange) flap was described to minimize the damage and dissection of the rectus abdominus muscle during the flap harvest. In the APEX flap, deep inferior epigastric vessels are harvested, but the abdominal wall structures are preserved by temporarily dividing the perforator or pedicle and reconstructing them at the end of dissection. Once outside the patient, the ligated vessels are microanastomosed [18]. This technique is recommended when more than one-third of the muscle belly or thickness could be lost, or two or more motor branches would be divided during isolation, especially in cases of lateral row perforators [18]. While this technique preserves the rectus abdominis muscle, it is very technically challenging and time-intensive to perform additional microanastomosis. Therefore, complex surgical cases like this benefit greatly from a co-surgeon model, which allows for shorter operative time [19].

#### 1.1.3. Surgical Techniques: Double-Pedicled DIEP Flaps

Double-pedicle DIEP flaps have been described to overcome some limitations of the conventional DIEP flaps. In this technique, the entire abdomen is harvested with two pedicles to reconstruct a single breast mound [12]. Typically, patients requiring a significant amount of skin and soft tissue after radiation for unilateral breast reconstruction benefit from double-pedicled DIEP flaps or any variation in conjoined/stacked flaps [12]. Often, the cranial and caudal internal mammary vessels are recipients of the flap, but at times, it is required to use intraflap anastomoses for adequate blood flow [12,20]. This requires complex pre-surgical planning and significant technical expertise from the co-surgeons involved in the case [12,20].

#### 1.1.4. Surgical Techniques: PAP Flaps

With advances in microsurgery, thigh-based autologous breast reconstruction options became available for patients who are not ideal candidates for abdominally based flaps. The profunda artery perforator (PAP) flap was originally used for pressure sores until its use was first described for breast reconstruction by Allen et al. in 2012 [21,22]. This flap is located on the posterior medial thigh, approximately 1 cm below the gluteal crease. The shape of the flap is long and elliptical, with an average weight of 367.4 g [21,23]. The average size of the perforator for this flap has been reported to be 1.9 mm [24]. The PAP flap has become a second choice when DIEP flaps are not an option or when a patient does not prefer the abdomen as a donor site [25]. The PAP flap can be used in various configurations, including stacked PAP flaps for unilateral breast reconstruction and four-flap procedure, which utilizes bilateral PAP and DIEP flaps for bilateral breast reconstruction [26].

#### 1.1.5. Surgical Techniques: TUG Flaps

The transverse upper gracilis (TUG) flap is another form of thigh-based flap available to patients, first described in 1992 by Yousif et al. [27]. It varies from the PAP flap in its more anterior position, and the TUG flap involves harvesting part of the gracilis muscle. The vascular supply for a TUG flap is the medial femoral circumflex, with an average artery diameter of 1.6 mm [28]. While this flap avoids abdominal scars, the soft tissue volume can be limited. However, the advantage of the TUG flap is the high plasticity of the tissue, which is more moldable than abdominal flaps and significantly more moldable than gluteal flaps [28]. This feature makes the TUG flap ideal in cases of skin-sparing mastectomy in women with small to medium breast sizes [28].

#### 1.1.6. Surgical Techniques: LAP Flaps

In 2003, de Weerd et al. first described the use of the lumbar artery perforator (LAP) flap for breast reconstruction. The LAP flap is supplied by the lumbar perforators at L3 and L4, where they run posterior to the psoas major muscle [29]. On average, the LAP flap has perforators with a diameter of 2.1 to 2.8 mm [30]. It is predicted that flaps as large as 21 × 12 cm may be harvested with flap weights reported as high as 750 g [30]. The location of the scar for a LAP flap is able to be hidden below the waistline and found to be satisfactory to patients [31]. However, the use of this flap is limited to experienced microsurgeons for several reasons. First, the flap needs to be harvested in a prone position due to its location, and the flap undergoes significant ischemia time as the patient needs to be flipped to a supine position for microanastomosis [29]. In addition, the LAP flap has a very short pedicle, which often requires the use of a vascular interposition graft to lengthen the length of the pedicle [12,29,32]. Due to the risk of prolonged ischemia times and the potential need for vascular interposition graft, the LAP flap often requires a well-orchestrated microsurgical team [33].

#### 1.1.7. Surgical Techniques: Four-Flaps

The most complex type of autologous breast reconstruction is a four-flap procedure. This involves reconstructing a patient’s breast with bilateral stacked flaps, which requires significant technical expertise. Typically, four-flap procedures are performed in patients with a lack of adequate soft tissue in one donor site to reconstruct the desired size of breast mounds [12]. The four-flap procedure is typically performed with bilateral DIEP and PAP flaps. The PAP flap is typically placed at the inframammary fold, while the DIEP flap is placed superiorly to restore the superior pole of the breast [12]. If harvesting flaps from the thigh is not an option, the LAP flap can be used in combination with the DIEP flap. 

### 1.2. Steps of Building Complex Autologous Breast Reconstruction Program 

#### 1.2.1. Phase 1—Establishing the Co-Surgeon Model

##### Infrastructure

**Figure 1:**
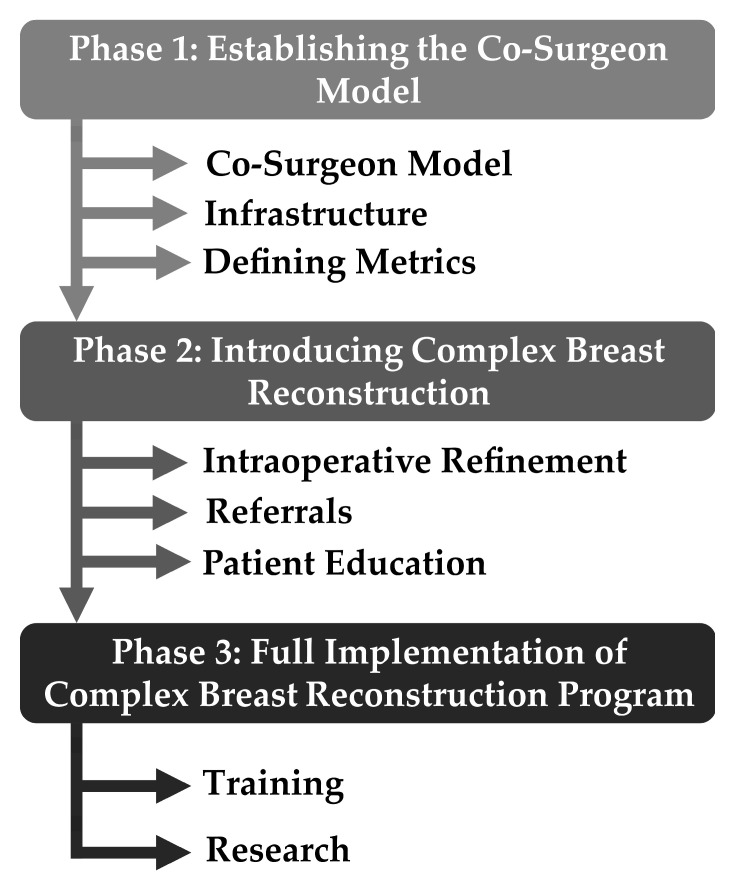
Roadmap of building a complex autologous breast reconstruction program.

The division of Oncologic Plastic Surgery at The Ohio State University Comprehensive Cancer Center—The James—has a total of seven microsurgeons. Of these, six microsurgeons specialize in breast cancer reconstruction. In order to build a complex autologous breast reconstruction program, it is critical to build a multi-disciplinary team that specializes in the care of breast cancer reconstruction patients. We first began by introducing the co-surgeon model in which two or more microsurgeons operate simultaneously to decrease operative time and increase efficiency (Figure 1). Typically, microsurgical autologous transfer requires multiple operative steps, including the following: (1) preparation of recipient chest vessels; (2) flap elevation; (3) flap harvest; (4) microsurgical anastomosis between recipient and donor vessels under microscope; (5) inset of flaps; and (6) closure of donor sites. Given the significant number of operative steps, the co-surgeon model was introduced and has been a widely accepted practice for bilateral autologous breast reconstruction in the United States. Studies have shown that this model led to a decrease in operative time and complication, suggesting a synergistic effect [34,35,36,37]. 

Therefore, we started co-surgeon practice during phase 1 to create teams of efficient microsurgeons who can perform complex autologous flaps. Due to the lack of a robust co-surgeon model at The Ohio State University, we allowed 3 months for the integration of phase 1. This accounted for a few roadblocks: (1) effective scheduling of two microsurgeons; (2) preparation of the operating room team for a two-team set-up with different surgeon preferences; and (3) time period to assess the efficacy and efficiency of the model. As surgery schedulers, operating room team, anesthesia team, and trainees are not familiar with the co-surgeon model, we held multiple team conferences and education sessions to allow for an easy transition into this model. As our institution is a teaching hospital, the co-surgeon model had a team of two attending microsurgeons, one microfellow or senior resident, and one junior resident. Typically, one attending surgeon and one junior resident dissected the chest recipient vessels while the other team harvested the flaps. All patients were placed in the breast reconstruction-specific Enhanced Recovery After Surgery (ERAS) protocol, including preoperative counseling, standardized anesthetic regimen, multi-modal pain regimen, and early mobilization to achieve early recovery and decrease prolonged hospitalization [38]. 

##### Outcomes Metrics

As part of our research focus, we recorded objective metrics that could be used for identifying areas of success and areas of improvement. These factors include many items such as flap size, perforator size, ischemia time, techniques used during surgery, complication data, hospitalization data, and other factors necessary to assess our outcomes.

#### 1.2.2. Phase 2—Introducing Complex Autologous Breast Reconstruction

##### Intraoperative Refinements

During phase 2, we first assessed the pitfalls and success of performing the co-surgeon model for autologous breast reconstruction. Due to scheduling conflicts, we first had pairs of microsurgeons that were matched based on their availability. However, we recognized that different microsurgeons with multiple backgrounds have different surgical preferences and approaches toward an operation. Therefore, we developed two surgical teams that consistently worked together to increase team efficiency. 

Once we had two consistent microsurgical teams, we then proceeded to introduce complex autologous breast reconstruction flaps. As the operating room staff was not familiar with specific operating room set-ups of these flaps, multiple team conference was held to discuss the following: (1) PAP flaps—a frog-legged, supine position set-up with bilateral lower extremity prepping; (2) LAP flaps—multiple position changes from supine to prone to supine, prepping of different body site per position change, and set up of back table microanastomosis between the flap and interposition grafts; and (3) four-flap—prepping of both abdomen and thighs. 

##### Referrals

Traditionally, patients who are not ideal candidates for DIEP flaps but require or desire autologous reconstruction due to radiation or patient preferences underwent latissimus dorsi flaps with implant [20,39]. As this method of reconstruction was the gold standard in patients with thin body habitus, other practitioners are not familiar with other complex autologous reconstruction options for these patients. Therefore, phase 2 was dedicated to the introduction of the complex autologous breast reconstruction program to other specialties, patients, and microsurgeons while performing these flaps in indicated patients. 

We first aimed to increase internal referrals by introducing the program during grand rounds of OSU’s Stefanie Spielman Comprehensive Breast Center and its affiliated hospitals. This allowed for other specialties to be familiar with the program and its referral process. In addition, we collaborated with the informational technology team to ensure that internal referral processes were in place and these referred patients were seen by the specialist teams. Subsequently, the referral processes were expanded to receive external referrals by increasing the community outreach to physicians practicing in regional cities and neighboring states. Similar to the internal referral process, grand rounds and regional conferences were utilized as the platform to share our program. 

##### Patient Education

To increase the use of complex autologous flaps for breast reconstruction, it is important to engage patients in comprehensive patient education. Our institution has over 15 different patient education pamphlets totaling approximately 40 pages, specifically on autologous breast reconstruction. These pamphlets include many additional high-quality external resources to further educate patients. 

#### 1.2.3. Phase 3—Full Implementation of Complex Autologous Breast Reconstruction

##### Training

Currently, our institution has one microsurgeon who was trained to perform all types of complex autologous breast reconstruction. While it is feasible to perform these types of flaps without prior training, the efficiency and efficacy of operation significantly increase with previous experience. Therefore, we are currently performing complex autologous breast reconstruction as the co-surgeon model with the pairing of experienced and non-experienced microsurgeons to increase the efficiency of the operation and to allow the partnering microsurgeon to develop an extensive understanding of nuances, pearls, and pitfalls of each complex autologous breast reconstruction.

In addition, our institution has a robust microsurgery fellowship program and plastic surgery residency program. Given that there is a limited number of institutions that specialize in complex autologous breast reconstruction, microsurgery fellows have a unique opportunity to learn and perform these procedures. Resident involvement in microsurgery is incredibly important for the education and safety of the patients [40,41]. As a team, microsurgery fellows and residents are an integral part of the complex autologous breast reconstruction program: junior residents on the recipient team and senior residents and microsurgery fellows on the flap harvest team. We plan to expand our program’s impact by training individuals who will be key stakeholders in building the complex autologous breast reconstruction program nationwide.

##### Research

We consistently recorded objective metrics to allow for assessment, improvement, and refinement of current program performance, patient access, and trainee education. We are currently in the process of implementing the BREAST-Q to incorporate patient-reported outcomes, which may shed light on the ways to improve patient outcomes in complex autologous breast reconstruction [42,43].

## 2. Materials and Methods

After obtaining institutional review board approval (Institutional Review Board of The Ohio State University; #2019E0643), we performed a chart review of the electronic medical record of patients who underwent complex autologous breast reconstruction flaps from 1 September 2020 to 1 August 2023. We divided this time period into four phases: phase zero; phase one; phase two; and phase three. The first surgical case of the program occurred on 22 September 2021. Phase zero was defined by the year before the founding of the complex autologous breast reconstruction program and served as a reference year for comparisons (from 1 September 2020 to 20 September 2021). Phase one represents the time needed to establish the use of the co-surgeon model for DIEP flaps (from 22 September 2021 to 31 December 2021). Phase two and phase three are the first and second years of offering complex autologous reconstruction options (from 1 January 2022 to 31 December 2022 and from 1 January 2023 to 1 August 2023, respectively).

Retrospective chart review was conducted to collect data on patient characteristics (age, body mass index, and comorbidities), intraoperative flap data (type of flap, weight, ischemia time, procedure time, and hospital length of stay), and complications. Once the data from the chart review were collected, we utilized Excel (Microsoft 365, Redmond, WA, USA) and Prism (version 10.1.0, Boston, MA, USA) to process the data and run statistical analyses. 

## 3. Results

In total, our program has reconstructed 74 breast mounds in 46 patients using 87 flaps (Table 1). Thirty-two patients underwent DIEP flaps, while 14 patients underwent complex autologous breast reconstruction (Table 1). Of the complex breast reconstruction patients, PAP flaps were most commonly performed, followed by LAP flaps (Table 1). Patients in the complex breast reconstruction group had lower BMI and lower rates of smokers, but the results were not statistically significant (Table 2). 

### 3.1. Demographics

There was no statistical difference between patients who received DIEP reconstruction versus complex reconstruction for age (52 yr. vs. 49 yr., *p* = 0.58), BMI (30.1 vs. 28.6, *p* = 0.33), length of stay (3.7 days vs. 3.7 days, *p* = 0.87), smoking history (43.7% vs. 21.4%, *p* = 0.15), radiation history (51.7% vs. 35.7%, *p* = 0.48), chemotherapy history (78.1% vs. 64.3%, *p* = 0.33), diabetes mellitus history (3.1% vs. 7.1%, *p* = 0.51), hypertension (18.8% vs. 0%, *p* = 0.08) and American Society of Anesthesiologist status (2.3 vs. 2.4, *p* = 0.53)

### 3.2. Phase Zero

**Figure 2:**
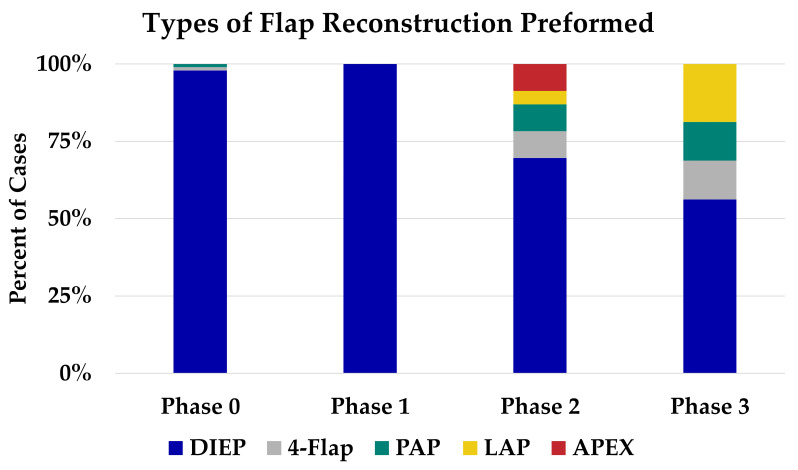
Trends in types of autologous breast reconstruction in our program. DIEP, deep inferior epigastric artery; PAP, profunda artery perforator; LAP, lumbar artery perforator; APEX, abdominal perforator exchange.

**Figure 3:**
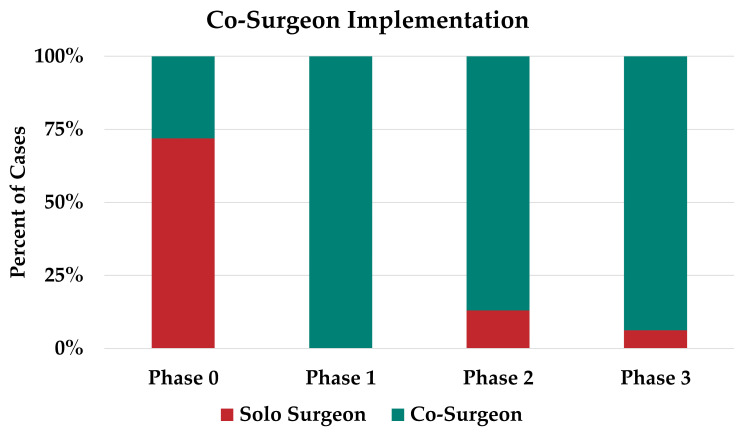
Trends in single and co-surgeon cases in our program.

During phase zero, a total of 136 breast mounds were reconstructed in 96 patients using 140 flaps. The DIEP flap accounted for nearly all of the reconstruction (98%), with a single case of four-flap and a single case of bilateral PAP flap reconstruction (Figure 2). Seventy-two percent of cases during this period were performed with a solo surgeon model (Figure 3). 

### 3.3. Phase One

During phase one, focus was placed on the introduction of the co-surgeon model for DIEP flaps. In this phase, 100% of cases were performed using the co-surgeon model, which lasted from 22 September 2021 to 31 December 2021 (Figure 1). This phase was dedicated to maximizing efficiency and building a team, and all reconstructions in this time period were DIEP flaps (Figure 3). The average operative time for DIEP flaps decreased by 122 min from an average of 638 min in phase zero (*p* = 0.05).

### 3.4. Phase Two

In phase two, we focused on expanding the use of complex autologous flaps. During this phase, the proportion of complex autologous flaps increased from 0% to 30% of cases (Figure 3). During this period, two four-flaps, two PAP flaps, one LAP flap, and two APEX flaps were performed (Figure 3). 

### 3.5. Phase Three

As the program continued to expand, we reached phase three, the full implementation of complex autologous breast reconstruction, 15 months after founding the program. During phase three, we continued the co-surgeon model, except when unilateral non-complex autologous breast reconstruction was performed (6%). Given the increased familiarity with preoperative planning, intraoperative set-up, and postoperative care, we increased the number and complexity of the flaps performed. During this period, complex reconstruction accounted for 44% of cases (two four-flaps, two PAP flaps, and three LAP flaps).

### 3.6. Program Effect on Surgical Times

Overall, there was a decrease in median surgical time for bilateral reconstruction of 124 min (652 min vs. 528 min, *p* = 0.03) after the creation of the program. The average surgical time decreased for bilateral co-surgeon (68 min, *p* = 0.05) and bilateral DIEP reconstruction (77 min, *p* = 0.01). After the start of the program, there has been an increase in co-surgeon unilateral breast reconstruction due to the rise in the complexity of unilateral reconstruction, such as stacked PAP flaps and LAP flaps. For stacked PAP flap cases, two PAP flaps were harvested to create one breast mound. Both flaps are microanastomosed to the internal mammary vessels using the anterograde and retrograde vessels. Due to the increase in the technical demand for these flaps, co-surgeon unilateral complex reconstruction took, on average, 112 min longer than co-surgeon unilateral DIEP reconstruction (*p* = 0.016). For overall complex reconstruction, there was a decrease of 184 min in the median operation time and 134 min in the mean. However, there were only two complex reconstructions in our control period prior to program creation, so statistical significance was not noted (*p* = 0.15). 

### 3.7. Complications

There was no statistically significant difference in the overall complication rate for the DIEP flaps vs. complex flaps (18% vs. 17%, *p* = 0.92) (Table 3). The most common complication was take-backs (seven flaps, 8%) (Table 3); the most common cause of take-backs was due to venous congestion (three flaps). However, our salvage rate was 100% during the hospitalization, and there was one single DIEP flap failure on postoperative day 15 due to a fall onto the flap. For four-flap reconstruction, the most common complications were take-backs occurring twice, hematoma occurring once, and pneumonia occurring in one patient (Table 3). We did not encounter any complications with PAP flaps and APEX flaps. Lumbar artery perforator flaps had one case of breast infection and one case of seroma (Table 3). The Clavien–Dindo classification for the complications showed that the majority of complications required surgical intervention under general anesthesia. Ninety percent (nine patients) of the 3b complications were due to take-backs, with 10% (1 patient) due to flap failure from vessel avulsion. We have no type 1, 4, or 5 complications. The type 2 complication was medication given for an infection (Table 4).

## 4. Discussion

Autologous breast reconstruction is the gold standard for providing natural, long-lasting breast reconstruction that often leads to higher patient satisfaction and aesthetic outcomes [4]. While the DEIP flap is the most commonly performed flap for autologous breast reconstruction, many patients are not ideal candidates for DIEP flaps due to low BMI, previous abdominoplasty, or the need for additional soft tissue [12,13]. Traditionally, these patients would undergo a combined autologous breast reconstruction such as latissimus dorsi flap and implant. However, this procedure still uses a prosthetic, which carries the risk of implant failure, infection, capsular contracture, and the need for implant exchange. Therefore, plastic surgeons have developed multiple novel techniques, such as multiple free flaps, using stacked flaps from non-abdomen donor sites. Despite the potentially higher patient satisfaction and aesthetic outcomes from complex autologous breast reconstruction, there is a limited number of centers offering this type of reconstruction due to the technically demanding nature of these procedures, longer operative time, and need for multiple microsurgeons [14]. With the increased concerns over implants due to breast implant-associated anaplastic large cell lymphoma, there has been a growing need for autologous breast reconstruction, and it is critical to build a center with a complex autologous breast reconstruction program.

Our preliminary experience of implementing a complex autologous breast reconstruction program shows that this program can be successfully established using a multi-phase approach. Since the start of our program, a total of 74 breast mounds have been reconstructed in 46 patients using 87 flaps. Over 23 months, there was a decrease in median surgical time for bilateral reconstruction by 124 min (*p* = 0.03), an increase in the number of co-surgeon cases by 66% (*p* < 0.01), and an increase in the number of complex autologous breast reconstruction by 42% (*p* < 0.01). The co-surgeon model was instrumental to the success of building this program as it allowed microsurgeons to adapt to new techniques and build team efficiency [14,19,35]. Two co-surgeon teams consisted of one junior microsurgeon experienced with complex flap breast reconstruction and one senior microsurgeon with at least 5 years of practice with no prior experience. By allowing the combination of years of technical excellence and familiarity with complex flaps, we were able to perform 30 complex flaps in 14 patients during 23 months of the program. While our average operative time of complex flaps (568 min) is higher than the published range of 520.7 to 610.3 min, we believe that operative time will continue to improve as microsurgeons, operating room staff, and trainees gain more experience [6,44].

Despite the infancy of our program, we have performed a wide range of complex autologous breast reconstructions, including unilateral stacked PAP flaps, bilateral PAP flaps, LAP flaps, four flaps using bilateral DIEP/PAP flaps, four flaps using bilateral DIEP/LAP flaps, and APEX flaps. The range of operative time was from 398 to 719 min, and the range of hospitalization was from 3 to 5 days. The most common complication in complex autologous breast reconstruction was take-back (7% vs. 8% in DIEP flaps). However, this only occurred in four-flap patients, which was most likely due to increased complexity and having flaps buried in stacked flaps. To maximize the aesthetic appearance of the reconstructed breasts, we frequently bury flaps in stacked flap breast reconstruction. While other monitoring mechanisms, such as implantable Doppler, are placed, it is challenging to fully assess the flap when the flap cannot be visualized [45]. Therefore, we have a low threshold for taking our complex flaps back to the operating room for exploration and potential flap salvage as needed. The majority of our flap take-backs were due to venous congestion secondary to pedicle positioning. To allow for stacked flap configuration, flap pedicles are placed in a specific configuration (anterograde anastomosis of a caudally placed flap and retrograde anastomosis of a cranially placed flap using internal mammary vessels or thoracodorsal vessels) within a breast pocket [46]. Given that flap pedicles must cross, it is critical to place the pedicles in an orientation that the pedicles will not kink or compress. Similarly, Haddock et al. found that stacked flap reconstruction has a higher rate of flap take-back but similar rates of flap failure rate between single and stacked flap breast reconstruction [6]. In our experience, all complex flaps were salvaged despite the higher flap take-back rate.

In addition, our program was able to successfully increase the number of complex flaps while maintaining a similar complication profile as single-flap breast reconstruction. Despite the increased number of donor sites in patients undergoing complex autologous breast reconstruction, the rate of donor-site complications was similar between DIEP and complex flaps except for the rate of seroma in LAP patients (one out of five donor sites). Studies have shown that the rate of seroma was higher than in traditional donor sites, and we have begun to perform more aggressive donor site closure and use of compression to decrease the rate of seroma [31]. Interestingly, our length of stay in complex flaps stayed similar to patients undergoing DIEP flaps except for the four-flap patients. As these patients stayed an average of 1 to 2 days longer than other complex flaps, we believe that this finding is secondary to the pain and difficulty with mobilization due to multiple-donor sites. In addition, the length of operative time is longer in these patients, and studies showed that there is a 27% increase in the risk of a postoperative complication for every additional hour of operative time in bilateral autologous breast reconstruction [35]. Therefore, one of our goals is to decrease the operative time in four-flap patients by 60 min by the end of phase 3.

Future directions to this program include (1) expanding the co-surgeon model, (2) increasing patient access through dedicated training, and (3) refining the program with our research findings. Currently, the main co-surgeon model that we use involves both surgeons being present for the entirety of reconstruction. However, this type of scheduling requires the co-surgeon to forego an entire operative day when they could have performed additional operative cases. As our operative experience grows and the program becomes busier, we plan to transition our co-surgeon model to a model when co-surgeons assist in two staggered cases during the key portion of the case. Studies have shown that this model can be safely performed while decreasing the operative time, length of stay, and wound complications [14,35]. In this advanced co-surgeon model, we can decrease the average wait time for the operation and increase patient access. We anticipate that with the addition of new microsurgeons who have significant training in performing complex autologous breast reconstruction, we will be able to implement the advanced co-surgeon model.

Secondly, we will focus our efforts on increasing patient access through dedicated training of microsurgery fellows. For our program, the key factors of successful development included a high-volume center, experienced microsurgeons, multi-disciplinary collaboration, and a microsurgeon who is well-experienced in performing complex autologous breast reconstruction. We believe that having one microsurgeon who is well-versed with these types of procedures leads to multi-magnitude effects on patient care by greatly expanding breast reconstruction options to patients with an insufficient single-donor site, irradiated patients with significant pliable skin requirement, and patients with failed implant-based breast reconstruction to obtain a natural, ptotic breast. While Bodin et al. showed that a minimum of 50 cases are needed to be proficient in DIEP flaps, we believe that it would take a lesser number of flaps to become proficient in complex autologous breast reconstruction as the principle of perforator dissection and flap harvest does not change [47,48]. Therefore, we believe that one year of the microsurgery fellowship program would be sufficient to train and increase proficiency in microsurgery fellows. As evident from our program, it is critical to increase the number of these microsurgeons who can team up with their partners to sustain continued relationships with our center and educate future microsurgeons, which will ultimately increase patient access nationwide.

Lastly, we believe that outcome research is critical to the continued development and refinement of the program. Since the beginning of our program, we have instituted multiple protocols based on outcome metrics. We collected various perioperative metrics, including the following: (1) preoperative—referral patterns, patient characteristics, preoperative planning using computed tomography angiography (CTA), co-surgeon scheduling, and team efficiency; (2) intraoperative—operative time, intraoperative set-up time, co-surgeon involvement, and trainee participation; and (3) postoperative—flap monitoring, nursing staff training, and specific postoperative protocols. During the first three phases, we first focused our efforts on maximizing preoperative and intraoperative metrics to minimize complications. We have incorporated preoperative CTA for preoperative planning, intraoperative indocyanine green (ICG) angiography to evaluate the flap perfusion, use of multi-phasic bovie cautery, coordinated position change protocol, and development of dedicated flap recipient/harvest teams [20,49].

With these refinements, we were able to further decrease operative time and increase our flap success rate. However, we have noticed increased challenges in flap monitoring protocol due to the variations in the nursing staff. In contrast to a dedicated operative team and outpatient nursing staff, inpatient nursing team members change frequently, and not all members are experienced with additional flap monitoring devices such as implantable Doppler or pulse oximetry devices. Therefore, we plan to hold regular in-services discussing our program and modify our protocols as needed.

Limitations of our study include the small size of our study population. The sample size for statistical analysis is limited by the infancy of our program (<2 years of complex reconstruction) and by the fact that complex reconstruction is only suitable for certain patients. For example, we were not able to statistically analyze specific complication rates for each flap type due to the rarity of complications and small numbers of each type of complex reconstruction. Prospective data collection and expansion of our surgical team will allow us to overcome these limitations in the future.

## 5. Conclusions

Despite the growing need for autologous breast reconstruction using complex flaps, it has been challenging to increase the number of centers that provide these unique options to patients due to technical demand and multilevel collaboration. In this study, we have found that a complex autologous breast reconstruction program can be successfully established using a multi-phase approach, including the development of a robust co-surgeon model, and a dedicated program leads to increased patient access, decreased operative time, decreased length of stay, and enhancement of trainee education. We hope that the pearls, lessons, and challenges that we have faced will serve as a useful guide to those who wish to incorporate this program as a part of their practice.

## Figures and Tables

**Table 1:** Characteristics of flaps.

	Flaps (n)	Patients (n)	Average Ischemia Time (min)	Weight (Grams)	Surgical Time (min)	Length of Stay (Days)	BMI
**DIEP**	57	32	73	669	523	3.7	30.2
**4-Flap**	16	4	76	429	719	5.0	26.8
**PAP**	7	4	75	310	458	3.0	28.6
**LAP**	5	4	144	945	613	3.5	30.2
**APEX**	2	2	58	549	398	3.0	29.0
**Total**	87	46	78	629	537	3.7	29.7

DIEP, deep inferior epigastric artery; PAP, profunda artery perforator; LAP, lumbar artery perforator; APEX, abdominal perforator exchange; BMI, body mass index.

**Table 2:** Clinical characteristics of the program population.

Characteristic	Overall	DIEP	Complex	*p*
Number of Patients	46	32	14	
Number of Flaps	87	57	30	
Number of Breast Mounds Reconstructed	74	55	19	
Age (years)	51 (25–70)	52 (25–70)	49 (33–63)	0.58
BMI	29.6 (17.5–40.9)	30.1 (17.5–39.4)	28.6 (21.2–40.9)	0.33
Bra Cup Size (Self-Reported)				
A	4.3%	6.3%	0%	
B	13%	9.4%	21.4%	
C	52.2%	59.4%	35.7%	
D	8.7%	3.1%	21.4%	
≥DD	17.4%	21.9%	7.1%	
Unknown	4.3%	0%	14.3%	
Length of Stay (days)	3.7 (2–7)	3.7 (2–7)	3.7 (3–6)	0.87
Comorbidities				
Smoking				
Never	63%	56.3%	78.6%	0.15
Current	2.2%	3.1%	0%	0.50
Former	34.8%	40.6%	21.4%	0.21
Radiation History	46.5%	51.7%	35.7%	0.48
Chemotherapy History	73.9%	78.1%	64.3%	0.33
Diabetes mellitus	4.3%	3.1%	7.1%	0.54
Hypertension	13%	18.8%	0%	0.08
ASA Physical Status	2.3 (2–3)	2.3 (2–3)	2.4 (2–3)	0.53

DIEP, deep inferior epigastric artery; BMI, body mass index; ASA, American Society of Anesthesiologists.

**Table 3:** Characteristics of complications.

	Infection (%)	Take-Back (%)	Seroma (%)	Hematoma (%)	Wound Dehiscence (%)	Fat Necrosis (%)	Flap Failure (%)	Deep Vein Thrombosis (%)
**DIEP**	0	9	0	2	2	2	2	3
**4-Flap**	0	13	0	6	0	0	0	0
**PAP**	0	0	0	0	0	0	0	0
**LAP**	20	0	20	0	0	0	0	0
**APEX**	0	0	0	0	0	0	0	0
**Overall**	1	8	1	2	1	1	1	2

DIEP, deep inferior epigastric artery; PAP, profunda artery perforator; LAP, lumbar artery perforator; APEX, abdominal perforator exchange.

**Table 4:** Clavien–Dindo classification of complications.

	0 (n)	1 (n)	2 (n)	3a (n)	3b (n)	4 (n)	5 (n)
**DIEP**	24	0	1	0	7	0	0
**4-Flap**	1	0	0	0	3	0	0
**PAP**	4	0	0	0	0	0	0
**LAP**	2	0	0	2	0	0	0
**APEX**	2	0	0	0	0	0	0
**Overall**	33	0	1	2	10	0	0

DIEP, deep inferior epigastric artery; PAP, profunda artery perforator; LAP, lumbar artery perforator; APEX, abdominal perforator exchange.

## Data Availability

The data presented in this study are available on request from the corresponding author. The corresponding author will ensure that individual privacy and IRB compliance are not compromised during the transfer of datasets.
